# Influence of the COVID-19 Pandemic on Italian LGBT+ Young Adults’ Mental Health: The Role of Neuroticism and Family Climate

**DOI:** 10.3390/ijerph192315795

**Published:** 2022-11-27

**Authors:** Marina Miscioscia, Mikael Poli, Alessio Gubello, Alessandra Simonelli, Michela Gatta, Jorge Gato, Paola Rigo

**Affiliations:** 1 Department of Developmental Psychology and Socialization, University of Padua, 35131 Padua, Italy; 2 Department of Women’s and Children’s Health, University of Padua, 35128 Padua, Italy; 3 Faculty of Psychology and Education Sciences, Université Libre de Bruxelles, 1050 Brussels, Belgium; 4 Faculty of Psychology and Education Sciences and Center for Psychology, University of Porto, Rua Alfredo Allen, 4200-135 Porto, Portugal

**Keywords:** COVID-19, LGBT+ mental health, psychological well-being, young adults

## Abstract

Vulnerable populations have been among the most affected by the social consequences of the COVID-19 pandemic; among those, young people and sexual and gender minorities have seen their situation exacerbated by new specific regulations. The aim of the present study was twofold: first, to assess the role of family climate, concerning participants’ LGBT+ status during lockdown restrictions, in mediating the impact of the COVID-19 pandemic on personal quality of life and mental health (stress, depression, and anxiety); second, to assess how individual stable traits can moderate the relationship between the individual impact of COVID-19 on mental health outcomes. A total of 407 young adults aged 18 to 35 (M age = 25.03 years; SD = 4.68) who self-identified as being part of a sexual or gender minority took part in this study. Results highlight the association between negative family climate and internalizing symptoms of psychological distress, and its role as a partial mediator of the relationship between the impact of the COVID-19 pandemic at the individual level and mental health outcomes. Additionally, low personality trait levels of neuroticism significantly decreased the strength of the relationship between LGBT+ status during blocking restrictions and internalizing symptoms.

## 1. Theoretical Background

Around two years have passed since the COVID-19 pandemic started to spread around the world, and studies, spanning from social and educational science to physical and mental health research, have accumulated evidence of the consequences of the restrictive measures put in place to stop or slow down the transmission of the SARS-CoV-2 virus [1]. Among the most impactful restrictive measures, numerous nations—including the Italian government—ordered the total or partial closure of work, educational and public places, and a state of quarantine for all citizens.

In Italy, containment measures in the form of full lockdown restrictions for the whole population were mandated via a Legislative Decree [2] from 9 March to 4 May 2020 (also known as “Phase 1”), and included stay-at-home restrictions (with bans on leaving one’s own house and on traveling outside one’s own municipality unless strictly necessary, e.g., essential work or health emergencies), closure of schools and universities, sports facilities, and all non-primary-necessity businesses, and the introduction of evening curfew regulations. Such measures were progressively lifted until December 2020 [3], when they were reintroduced following a spike in COVID-19 cases, to then be eased again in different regions at different times starting from 22 April 2021, on the basis of the incidence of cases at the regional level [4].

In the context of actions aimed at containing the spread of epidemics, quarantine-based social isolation represented an important and significant environmental challenge that carried many adverse effects on the population’s psychological wellbeing [5,6,7,8,9,10]. The impact of the COVID-19 pandemic radically prevented interpersonal contact in almost all individuals’ contexts. Distance learning and teleworking and the impossibility of meeting in person with friends, partners, and potential romantic interests constituted critical challenges to individual psychosocial adjustment and wellbeing [11,12,13,14].

### 1.1. Prior Knowledge: Societal Stressors for LGBT+ People Living in Italy

Several international sources estimating LGBT+ acceptance within society indicate a generally unfavorable climate for sexual and gender minorities in Italy. In their Rainbow Europe 2022 map [15], the European region of the International Lesbian, Gay, Bisexual, Trans and Intersex Association (ILGA) reports that Italy is lagging behind in terms of achieving full equality for LGBT+ people. It occupies the 33rd place out of the 49 countries that were included in this statistic, scoring 24.76% on a scale from “Gross violations of human rights, discrimination” (0%) to “Respect of human rights, full equality” (100%) with respect to different aspects of legal and policy human rights management: equality and non-discrimination, family, hate crime and hate speech, legal gender recognition and bodily integrity, civil society space, and asylum. Furthermore, Italy’s latest available Global Acceptance Index (GAI, developed by the Williams Institute (UCLA) for the LGBTI Global Development Partnership) [16] is 6.94 on a scale from 0 to 10, indicating that overall, societal attitudes towards LGBT+ people, while not completely negative, can still improve. More recently, LGBT+ rights have been in the spotlight in Italy with debate surrounding the approval (still pending as of August 2021) of the anti-homotransphobia bill presented by Italian Chamber of Deputies member Alessandro Zan [17], arousing both positive and negative attention to sexual and gender minority people during a time of substantial challenges for the whole population.

Despite tentative steps forward in terms of legislation and policy, societal attitudes towards non-heterosexual people still represent a key element for the psychosocial well-being of SGM (Sexual and Gender Minority) groups that can produce detrimental outcomes for quality of life. The Organization for Economic Co-operation and Development (OECD) [18] highlights that Italy scored a 3.3 on a survey exploring acceptance of homosexuality on a scale from 1 (“Homosexuality is never justifiable”) to 10 (“Homosexuality is always justifiable”) in their latest “Society at a Glance” report, with the average for the 38 OECD member countries being 5.1. According to the latest European Union data report 2019 [19], only 68% of Italians agreed with the statement “Gay, lesbian and bisexual people should have the same rights as heterosexual people”, and 59% agreed with the statement “There is nothing wrong in a sexual relationship between two persons of the same sex”. Importantly, one-third (33%) would be completely uncomfortable if their child were involved in a romantic relationship with a person of the same sex. Overall, 69% felt that discrimination on the basis of sexual orientation was widespread in their country: in fact, Italy ranked 4th (out of 28 member states) for rate of perceived widespread discrimination against sexual minorities among the general population, with higher ranks indicating higher perceived diffuse inequality of treatment [19].

Regarding gender minority individuals, it was only in 2017 that the Italian Constitutional Court declared that sex reassignment surgery (SRS) was not mandatory to access legal gender identity recognition [20]. For those gender minority individuals who seek gender affirmation treatments, this process can be taxing in terms of availability of resources and time. Gender-affirmation clinics are scarce, particularly in the south, and are not present in all Regions [21]. Often, it takes several years between the initial request to undertake medical treatment and obtaining legal recognition of one’s gender identity (i.e., for local courts to allow name and gender marker rectification on identity documents) (see, for instance [22]). With regard to intersex people, the Italian legislation does not contemplate protection from non-consensual surgical interventions in childhood, a common practice that is still implemented today in most countries around the world despite its reported harmfulness [23,24,25], effectively hampering the possibility for self-determination and the right to bodily integrity and autonomy [26].

### 1.2. COVID-19 and Psychological Adjustment for LGBT+ People

The historically vulnerable segments of the population have been the most affected by the COVID-19 pandemic [9]. For example, disparities in both medical and psychosocial outcomes have been documented for Black, Asian, and other ethnic minority populations in the U.S. and Europe [27,28,29,30,31], as well as for sexual and gender minorities [32,33]. The pandemic has amplified the disparities between the LGBT+ community and the rest of the population, since the former suffer not only from financial and social complications, but also from consequences of the stigma associated with their LGBT+ identity [34,35]. Challenges brought about by the pandemic for LGBT+ people have included hampered access to clinical care, including HIV testing and treatment [36] and psychological support, and maltreatment and abuse within the household, as well as the impossibility of pursuing legal recognition of their gender identity [33,37,38]. In general, younger people appear to be more likely to experience higher levels of anxiety, depression, stress, and post-traumatic stress than older adults [9,10] and several studies have shown that the COVID-19 pandemic represented an additional stress factor for the psychosocial adjustment of LGBT+ young adults [39,40,41,42,43].

Regarding the Italian situation, Mattei et al. [44] observed that the economic recession amplified by the public health crisis may have further aggravated the burden on mental health for LGBT+ groups through heightened discrimination. The situation seems to have been aggravated in the case of transgender and GQNB (genderqueer/nonbinary) Italian people, due to higher stress levels due to living with non-accepting family members, perceived lack of support from LGBT+ circles, and difficulties in accessing health facilities and undertaking hormone replacement therapy [45].

### 1.3. Factors Associated with Psychological Adjustment during Pandemic Time

Diener [46] identified three macro areas of elements influencing subjective well-being (SWB): temperament and personality, values and goals, and cultural and societal factors. This last group of factors, in particular, can influence SWB through a variety of mechanisms related to a country’s ability to fulfill basic needs (food, clean water, and health) and to the community’s coping patterns, degree of regulation of individual desires, and social support, the latter particularly within families [46]. Well-being has been associated with how individuals negotiate and adapt to the developmental transitions of adult life throughout the life cycle [47,48], as well as with personality traits (neuroticism in particular) [49,50], parental support and family dynamics [51,52,53], and achievement of early career goals and paid workload [54,55].

Individual factors, such as aspects of the personality as conceptualized according to the Big Five model [56], play an important role in determining an adjustment to stressful situations; indeed, resilience was positively associated with trait extraversion and conscientiousness and negatively correlated with neuroticism, which entails low emotional stability [57,58]. Although the mechanisms through which neuroticism may affect psychological well-being are still debated [59], one possible explanation is that it may act through the cognitive appraisal of environmental demands. In this regard, Schneider [60] found both neuroticism and threat appraisals to be associated with negative emotional experiences and poor task performance, with the first predicting the second. High neuroticism levels may indeed predispose to worse psychophysical stress responses [61,62], especially when environmental demands are interpreted to be threatening and to exceed one’s own coping resources. So far, the literature has highlighted connections between neuroticism and various cognitive aspects of individuals’ experiences, such as rumination and worrying [63], time perspective [64], and cognitive emotion regulation strategies such as self-blame and catastrophizing [65,66], as well as environmental factors. Considering the latter, evidence suggests that values of a given culture could influence the level of neuroticism contributing to dysfunctional adjustment to life challenges [67,68].

The personal environment, especially the family and home context, represents a key factor influencing resilience and psychosocial adjustment throughout the lifespan [69,70,71]. Resilience has been described as a dynamic process that encompasses the ability to maintain psychophysical well-being when faced with adversities [72,73], favoring positive psychological functioning over time [74]. Connor and Davidson [75] conceptualize it as a measure of an individual’s stress coping ability; indeed, high levels of resilience are linked to better psychophysical outcomes, while lower levels are associated with negative mental health indicators such as depression and anxiety [76]. Importantly, resilience and adaptation can be influenced by psychosocial factors, such as personality traits, optimism, family functioning, and natural disasters (for a comprehensive account, see [70]), and while most are considered to be relevant at all ages, Noble and McGrath [71] report that some aspects of the environment, such as a positive family context and peer relationships, represent an important element for young people’s adjustment and psychosocial well-being. Indeed, although higher levels of psychosocial maladjustment have been reported by the global community at large following the outbreak of the pandemic, younger people appear to be more likely to experience higher symptoms of anxiety, depression, stress, and post-traumatic stress disorder than older adults [10].

In minority groups at risk of discrimination, not only within the socio-cultural context but also within the domestic environment [77], a deepening understanding of how low levels of affective resilience—a dimension of personality traits that reflects stable strategies to recover the homeostasis from dysregulated states of Self—can contribute as an additional threatening risk factor for individual mental health [78]. In an extremely complex context such as that in the wake of state-mandated social isolation and home confinement measures, particular attention should be paid to the influence that COVID-19 pandemic restrictions could have had on family interactions in LGBT+ young people’s experience, as family constitutes one of the most immediate sources of direct impact on an individual’s healthy development [79,80], as well as representing a key element that health professionals can focus on to promote psychological wellbeing in the population [81,82].

Among the LGBT+ community, young individuals were more likely to report higher symptoms of depression and anxiety during the COVID-19 pandemic and complained of being either uncomfortable at home, isolated from their heterosexual and cisgender friends, or not working [40,41,42,43,83,84]. Additionally, not attending higher education, experiencing the daily impact of the COVID-19 pandemic, and having a negative family climate were significantly associated with higher levels of internalizing symptoms [40]. However, research has shown that personality and personal environment can both exert an influence on an individual’s experience in different ways across different age ranges [85], favoring or hampering positive mental health outcomes in young adults’ adjustment to adverse situations [86].

Following on from the aforementioned, the present work aimed to assess the psychosocial correlates of the COVID-19 pandemic on Italian LGBT+ young adults to identify potential predictors of adverse mental health conditions. The first aim was to assess the role of family climate in relation to participants’ LGBT+ status during lockdown restrictions in mediating the impact of the COVID-19 pandemic on personal quality of life and mental health (stress, depression, anxiety). We expect to observe family climate to be a partial mediator between the impact of COVID-19 in terms of personal impact and depression, anxiety and stress [84]. In line with research on resilience in psychosocial adaptation to stressful events [58], the second aim was to assess how individual stable traits can moderate the relationship between the individual impact of COVID-19 on mental health outcomes. The literature confirms that low levels of neuroticism are associated with better wellbeing and low levels of mental health. We expect to observe a stable individual factor, i.e., trait neuroticism, to be a moderator.

## 2. Materials and Methods

### 2.1. Participants

Our sample included 407 participants (Mage = 25.03 years; SD = 4.68). Inclusion criteria were being 18–35 years of age, residing in Italy, identifying as being part of a sexual or gender minority, having disclosed one’s sexual or gender minority status to one’s family, having been in contact with one’s family during the lockdown and at the time of completion of the questionnaire, and having completed the questionnaire in its entirety. We collected data from 1294 participants; after applying inclusion criteria, 887 responses were excluded from our analyses. Participants who chose to freely describe their sexual orientation as they saw fit (using the dedicated space comprised in the questionnaire) included: biromantic asexual, homoromantic asexual, panromantic demisexual, demisexual, fluid, polyamorous pansexual, mostly heterosexual, queer, and questioning. Similarly, participants who indicated their gender identity as “other” described themselves as genderfluid, genderqueer, questioning, not defining, queer, and transgender non-binary. A total of 32.18% of participants reported previous health (physical or psychological) problems. Table 1 summarizes our sample’s main sociodemographic characteristics. 

### 2.2. Procedure

The present study is a part of the “Project Global Queerantine”, where data from seven European and Latin American countries have been collected with the aim of researching the social support networks and psychological health of LGBTQ+ individuals during the COVID-19 pandemic [40]. The research project was approved by the Ethics Committee of the University of Padova, Italy (Psychological Research Areas of the University of Padova n. 3591/2020); this research involving human participants was performed following the Declaration of Helsinki. The research protocol consisted of a 71-item questionnaire created and hosted on the online platform Qualtrics, which could be accessed through a web link. Participants were recruited via non-probabilistic convenience and chain sampling (through Facebook, Instagram, local and national LGBT+ associations, and word-of-mouth). Local and national LGBT+ associations were invited, with a letter from the Principal Investigator, to support and publicize the study by inviting their associates to participate (sending them an email with an endorsement to the research and the Qualtrics link). The associates were informed that the study was anonymous and not compulsory; they were also informed that, at the conclusion of data collection and analysis, the association was going to organize dissemination seminars with the research team. 

The first page of the questionnaire displayed an informed consent form describing the research project and main objective which the participants were asked to agree to; consent was expressed by clicking on the “next page” arrow in the bottom right corner of the page, which redirected to the first section of the questionnaire (sociodemographic variables), and could be withdrawn at any point during the study. Contact information for the Principal Investigator was provided should the participants need further clarifications during completion and, more generally, should they have any concerns or questions regarding the research project. 

All participants were informed of the anonymity of their answers unless they agreed to be contacted again for future studies by providing a personal email address: in this case, confidentiality was guaranteed, and participants were informed that all contact information would be available to the research team exclusively. In all cases, participants were informed that data would be analyzed on an aggregate and anonymous basis. 

Data were collected in two waves from 30 May 2020 to 21 April 2021. Wave one lasted from May 2020 to July 2020; wave two took place from December 2020 to April 2021.

### 2.3. Measures

#### 2.3.1. Psychosocial Effects of the COVID-19 Pandemic (Ad Hoc Questionnaire)

We administered 11 items relating to the psychosocial effects that the COVID-19 pandemic may have entailed specifically for LGBT+ young adults who were in contact with their family during the period of state-mandated restrictions (Table 1). All the items were originally devised in an ad hoc manner by Gato et al. [4] on the basis of a review of the literature and translated and adapted to the Italian context by our team by means of a back translation. To uncover the underlying structure of the Psychosocial Effects of the COVID-19 Pandemic section of the questionnaire, we replicated the procedure employed by the Portuguese research group [40,84]. Before proceeding, we reverse scored item 8 to align all numerical rating scales. We conducted a Kaiser–Meyer–Olkin (KMO) test, obtaining an overall KMO value of 0.778 for the remaining sample (n = 337), indicating moderate reciprocal correlation between the 11 items, which were therefore suitable for the EFA. The analysis yielded a 3-factor structure, explaining 48.1% of the total variance. The null hypothesis that three factors were sufficient in explaining the covariance among the variables was accepted (χ^2^ (25) = 30.14, *p* = 0.219). Items 4, 5, and 11 were removed from further analysis because of low correlation with the rest of the items: the communality values were 0.16, 0.04, and 0.13, respectively, and their highest rotated factor loadings were 0.37 (item 4), 0.18 (item 5), and 0.33 (item 11) (cut-off for inclusion ≥ 0.40). We then conducted a second EFA with the remaining 8 variables. A KMO value of 0.753 confirmed our data’s suitability for further analysis, and our EFA further substantiated the previous 3-factor structure, explaining 61.9% of the total variance. The model’s goodness of fit was tested, and the null hypothesis that three factors were sufficient in explaining the covariance among the variables was accepted, χ^2^ (7) = 13.06, *p* = 0.07. EFA results are reported in Table 2. We averaged the scores belonging to each factor in order to obtain three composite summary scores: for individual impact (factor 1) and social isolation (factor 2), higher values (range: 0–10) indicate a worse subjective experience [43]. For family climate (factor 3), after the second EFA we returned to the original raw scores for item 8, and reversed the scores for items 6 and 7, so that higher values of the composite score (range: 0–10) indicate a better subjective experience.

*Factor 1 (Individual Impact)* gathered all questions pertaining to the impact that the COVID-19 pandemic may have implied for participants at the individual level. The items included in this measure were:

▪ “To what extent has the COVID-19 pandemic affected your life?”
▪ “To what extent do you currently feel limited in carrying out your usual activities due to the COVID-19 pandemic?”
▪ “To what extent has the COVID-19 pandemic affected you emotionally (has made you feel angry, scared, upset or depressed)?”

*Factor 2 (Social Isolation)* gathered all questions relating to the degree of perceived isolation from peers due to state-mandated restrictions. The items included in this measure were:

▪ “To what extent has the COVID-19 pandemic made you feel isolated from your heterosexual or cisgender friends?”
▪ “To what extent has the COVID-19 pandemic made you feel isolated from your LGBT+ friends?”

*Factor 3 (Family Climate)* gathered all questions related to the participants’ subjective wellbeing as LGBT+ young adults in a family system while COVID-19 restrictions were in place. The items included in this measure were:

▪ “To what extent do you feel uncomfortable in your family in this specific moment of the COVID-19 pandemic?”
▪ “To what extent did you feel “suffocated” because you could not express your LGBT+ identity with your family when restrictions were in place?”
▪ “If the people you currently live with are aware of your LGBT+ identity. how do they respond to this?”

#### 2.3.2. NEO Five-Factor Inventory-3 (NEO-FFI-3)

The NEO-FFI-3 [87,88] provides a self-report measure of personality traits according to the Big Five theory of personality [56,89]. For the purposes of this study, we used a neuroticism scale with 12 items, each rated by the participant on a 5-point Likert scale ranging from 1 to 5, where 1 = “Strongly disagree” and 5 = “Strongly agree”, according to their pre-COVID-19 pandemic experience. Neuroticism scores were calculated by reverse scoring the appropriate items, summing all values to obtain raw scores (range: 12–60), computing z-scores, and finally calculating T-scores (M = 50, SD = 10) through a standardization process. Standardized coefficient alpha for Neuroticism was 0.87 (the Italian validation of the instrument reports α = 0.75 [88]). Because biological sex affects NEO-FFI-3 Neuroticism results but not much is known about the influence of gender identity, particularly in the case of gender variance, in calculating z-scores we applied the average and SD for the female population for our cisgender female participants, the average and SD for the male population for our cisgender male participants, and the total population average and SD for our non-cisgender participants.

#### 2.3.3. Enacted Stigma Scale of the Minority Stress Scale (MSS)

The Enacted Stigma dimension contained in the MSS [90], which we employed for control purposes, assesses the frequency of experienced stigma in the form of physical and verbal aggression, discrimination, and subjective feelings of societal exclusion (e.g., “Because of my sexual orientation I have been the target of verbal aggressions”). Originally validated for use with Italian gay and bisexual men and based on minority stress theory [35], we adapted it to include gender minorities as well (for example, the item above was rephrased as “Because of my sexual orientation or gender identity I have been the target of verbal aggressions”). The Enacted Stigma scale is composed of four items to which answers are given on a 5-point Likert scale ranging from 1 (“Never”) to 5 (“Always”). Enacted stigma scores were calculated by averaging the values of the 4 items composing the scale (range: 1–5).

#### 2.3.4. Depression, Anxiety and Stress Scale (DASS-21; for Mental Health Measures)

This self-report questionnaire [91,92] is designed to measure the level/intensity of internalizing problems over the previous week. The instrument is divided into 3 subscales (Depression, Anxiety, Stress) containing 7 items each. Participants were asked to indicate the extent to which each item reflected their experience using a 4-point Likert scale where 0 = “Did not apply to me at all”, 1 = “Applied to me to some degree, or some of the time”, 2 = “Applied to me to a considerable degree or a good part of time”, and 3 = “Applied to me very much or most of the time”. Items include statements such as “I was unable to become enthusiastic about anything”, “I felt I was close to panic”, and “I was intolerant of anything that kept me from getting on with what I was doing”. The standardized coefficient alpha for each 7-item scale was 0.92, 0.89, and 0.89, for Depression, Anxiety, and Stress, respectively. For their Italian community sample, Bottesi et al. [92] reported coefficient alpha values of 0.82, 0.75, and 0.85, respectively. DASS-21 scores were calculated by summing the values of the 7 items for each separate scale (range for all scales: 0–21).

### 2.4. Data Analysis

Data were analyzed using the statistical software R 3.6.3 [93], the integrated development environment RStudio 1.2.5003-1 [94] and jamovi 1.8 [95]. Figures were created using R 3.6.3 [93] and RStudio 1.2.5003-1 [94]. 

#### 2.4.1. Preliminary Analysis

To better describe the sample, we preliminary assessed the influence of *sex assigned at birth* (four levels: female, male, intersex, other), *gender identity* (four levels: cisgender, transgender, non-binary, other), and *sexual orientation* (four levels: homosexual, bisexual, pansexual, other) on depression, anxiety, stress and individual impact through three separate nonparametric Kruskal–Wallis one-way analyses of variance (ANOVA), and post hoc Dwass–Steel–Critchlow–Fligner (DSCF) pairwise comparisons. As control analysis for the influence of time (on measures of psychosocial well-being, we checked for differences in dependent variables between wave 1 and wave 2 participants through a nonparametric independent samples Mann–Whitney U test. 

#### 2.4.2. Multiple Regressions: Predictors of Adverse Mental Health Conditions

To identify potential psychosocial correlates of the COVID-19 pandemic as predictors of psychological distress measures (DASS), we ran three linear regression models with (M1) depression, (M2) anxiety and (M3) stress as our dependent variable (DV). All the models (M1, M2, and M3) included the same predictors and differentiated for the dependent variable. For each DV, we tested and compared fit for two multiple linear regression models through the adjusted R2 coefficient of determination. Model 1 included variables age, gender identity, sexual orientation, living situation, educational level, employment situation, relationship status, other medical and/or psychological conditions, enacted stigma, and wave (two levels: 1 = May–July 2020; 2 = December 2020–April 2021). Model 2 included all Model 1 variables with the addition of neuroticism and COVID-19-related variables, i.e., individual impact, family climate, and social isolation.

#### 2.4.3. Conditional (Moderated) Mediation Analysis: Mechanisms Underlying Psychological Distress during the COVID-19 Pandemic

To gain a deeper understanding of the psychosocial mechanisms underlying mental health in our sample, we computed three separate conditional (moderated) mediation models with four variables in jAMM; for three distinct DVs (depression, anxiety, stress), each model included individual impact as a covariate, family climate as a mediator and neuroticism as a moderator.

## 3. Results

### 3.1. Descriptive and Preliminary Analysis

We computed descriptive statistics (Table 3) for our sample to explore the characteristics of participants. 

Overall, according to the recommended cut-off scores for conventional severity labels, 33 participants reported moderate levels of depression, and moderate to severe anxiety and stress on average. Mean NEO-FFI-3 Neuroticism scores were over 2 SD above the mean for T-scores (M = 50, SD = 10), indicating high levels of trait neuroticism. MSS Enacted stigma scores indicate that, globally, participants reported experiencing sporadic episodes of aggression on the basis of their sexual orientation and/or gender identity.

We reported below the significant results of preliminary analyses assessing for differences between participants based on sex assigned at birth, gender identity, sexual orientation, and time of data collection.

*Sex assigned at birth*: Kruskal–Wallis ANOVA results indicated a statistically significant influence of sex assigned at birth on all DASS-21 scores (χ^2^ depression (3) = 18.11, *p* < 0.001, ε² = 0.05; χ^2^ anxiety (3) = 16.92, *p* < 0.001, ε² = 0.04; χ^2^ stress (3) = 23.96, *p* < 0.001, ε² = 0.06), ε² = 0.03). Epsilon-squared coefficients suggest a weak effect on anxiety and neuroticism, and a moderate effect on depression and stress. Post hoc DSCF pairwise comparisons indicated statistically significant differences between AMAB (assigned male at birth) and AFAB (assigned female at birth) participants in depression (W = −5.416, *p* < 0.001), anxiety (W = −5.207, *p* < 0.001), stress (W = −6.865, *p* < 0.001), and individual impact (W = −3.867, *p* = 0.032), all of which were higher for the AFAB group.

*Gender identity:* Kruskal–Wallis ANOVA results indicated a statistically significant although weak influence of gender identity on depression (χ^2^(3) = 8.32, *p* = 0.040, ε² = 0.02). DSCF pairwise comparisons indicated statistically significant differences between cisgender and non-binary participants for depression (W = 3.926, *p* = 0.028), with the non-binary group obtaining higher depression. 

*Sexual orientation*: Kruskal–Wallis ANOVA results also indicated a statistically significant influence of sexual orientation on measures of depression (χ^2^(3) = 18.12, *p* < 0.001, ε² = 0.05), anxiety (χ^2^(3) = 11.12, *p* = 0.011, ε² = 0.03), stress (χ^2^(3) = 14.22, *p* = 0.003, ε² = 0.04). The measure of effect size suggests a weak influence of sexual orientation on anxiety and stress, and a moderate influence on depression. Post hoc DSFC pairwise comparisons highlighted a statistically significant difference between bisexual and homosexual participants for depression (W = −4.497, *p* = 0.008), stress (W = −3.660, *p* = 0.047), and between homosexual and pansexual participants for depression (W = 4.623, *p* = 0.006), anxiety (W = 4.562, *p* = 0.007), stress (W = 4.420, *p* = 0.010). 

*Time *(*wave 1* vs. *wave 2*): Finally, to examine the influence of time on measures of psychosocial well-being (wave 1 vs. wave 2) Mann–Whitney U test indicated a small statistically significant influence of time of completion of the questionnaire on depression (U = 14564, *p* = 0.003, rrb = 0.18) anxiety (U = 14062, *p* < 0.001, rrb = 0.21), stress (U = 14351, *p* = 0.001, rrb = 0.20) and a moderate influence on individual impact (U = 9451, *p* < 0.001, rrb = 0.47). The wave 2 group reported higher scores in all variables.

### 3.2. Multiple Regressions: Predictors of Adverse Mental Health Conditions

Model 1 (M1 Depression; M2 Anxiety; M3 Stress) indicated that other medical and/or psychological conditions (F depression (2, 391) = 19.45, *p* < 0.001; F anxiety (2, 391) = 22.52, *p* < 0.001; F stress (2, 391) = 12.01, *p* < 0.001); enacted stigma (F depression (1, 391) = 15.40, *p* < 0.001; F anxiety (1, 391) = 20.58, *p* < 0.001; F stress(1, 391) = 10.45, *p* = 0.001); and time of completion of the questionnaire (F depression (1, 391) = 4.91, *p* = 0.027; F anxiety (1, 391) = 6.77, *p* = 0.010; F stress (1, 391) = 5.83, *p* = 0.016) significantly predicted all DASS-21 scores. Furthermore, the employment situation significantly predicted depression (F (1, 391) = 7.63; *p* = 0.006), while age significantly predicted anxiety (F (1, 391) = 9.77, *p* = 0.002) and stress (F (1, 391) = 4.97, *p* = 0.026) (Table 4). However, with the exception of other medical and/or psychological conditions and age, all predictors lost their statistical significance once the three COVID-19 scales and neuroticism were added to the model (Model 2: M1 Depression; M2 Anxiety; M3 Stress). More specifically, in Model 2, individual impact (F depression (1, 387) = 10.81, *p* = 0.001; F anxiety (1, 387) = 9.40, *p* = 0.002; F stress (1, 387) = 22.00, *p* < 0.001) and neuroticism (F depression (1, 387) = 143.83, *p* < 0.001; F anxiety (1, 387) = 49.97, *p* < 0.001; F stress (1, 387) = 65.24, *p* < 0.001) predicted all DASS-21 scores, while family climate (F depression (1, 387) = 7.92, *p* = 0.005; F anxiety (1, 387) = 11.32, *p* < 0.001) and other medical and/or psychological conditions (F depression (2, 387) = 4.22, *p* = 0.004; F anxiety(2, 387) = 8.92, *p* < 0.001) predicted depression and anxiety scores. Age was also reconfirmed as a predictor of anxiety (F (1, 387) = 7.65, *p* = 0.006).

Model 2 showed higher goodness-of-fit and prediction power than Model 1 for all the DASS-21 scales, explaining between 38 and 52% of the variance for all the DV scores, as indicated by adjusted R² values. 

### 3.3. Condition (Mediated) Moderation in jAMM: Mechanisms Underlying Psychological Distress during the COVID-19 Pandemic

**Figure 1:**
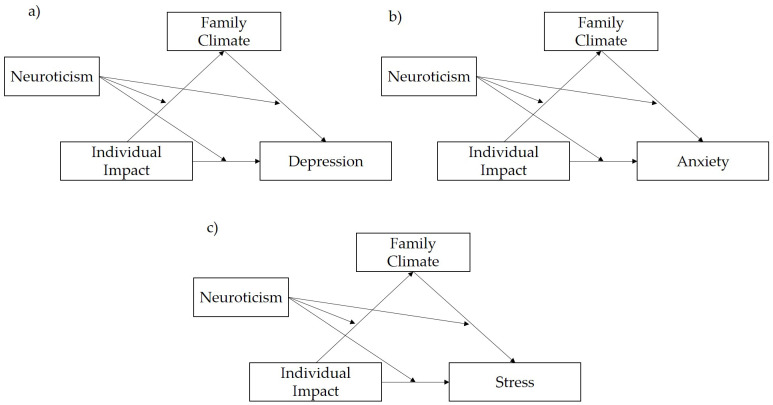
Moderated Mediation Models: (**a**) Depression; (**b**) Anxiety and (**c**) Stress.

*Depression.* Neuroticism did not moderate the path in a substantial way from individual impact to family climate (IE = −0.003, CI = [−0.010, 0.004], beta = −0.036, z = −0.781, *p* = 0.435) and family climate and depression (IE = 0.007, CI = [−0.005, 0.018], beta = 0.110, z = 1.174, *p* = 0.240) (Table 5; Figure 1a). However, neuroticism moderated the path from individual impact to depression, because the interaction between individual impact and depression is different from zero (IE = 0.017, CI = [0.004, 0.031], beta = 0.093, z = 2.538, *p* = 0.011).

We can see the mediation effect looking at the mean level of the moderator (Table 6). At the average mediated effect across levels of the neuroticism (mean level), family climate partially mediated the effect of individual impact on depression (IE= 0.066, CI = [0.010, 0.121], beta= 0.021, z= 2.31, *p* = 0.021). Moreover, the single components of the indirect path, the direct and total mediation were significant.

For high levels of neuroticism (mean + SD), the mediated effect of family climate to predict depression became smaller and did not reach the level of significance (IE = 0.048, CI = [−0.012, 0.108], beta = 0.015, z= 1.56, *p* = 0.118). Instead, the direct mediation of individual impact to depression showed a larger effect (IE = 0.516, CI = [0.276, 0.756], beta = 0.166, z = 4.21, *p* < 0.001).

For low levels of neuroticism (mean-SD), the mediated effect of family climate to predict depression maintained the level of significance (IE = 0.075, CI = [−0.010, 0.140], beta = 0.024, z = 2.25, *p* = 0.025). Instead, the direct mediation of individual impact to depression showed a smaller effect that did not reach the significance (IE = 0.253, CI = [−0.019, 0.525], beta = 0.081, z = 1.821, *p* = 0.069).

*Anxiety.* Neuroticism moderated the path from Individual Impact to Anxiety (IE= 0.025, CI = [0.010, 0.039], beta = 0.138, z = 3.389, *p* < 0.001). As in the previous model (depression), neuroticism did not moderate the path in a substantial way from individual impact to family climate (IE = −0.003, CI = [−0.010, 0.004], beta= −0.036, z = −0.781, *p* = 0.435) and family climate and anxiety (IE = 0.007, CI = [−0.020, 0.005], beta = 0.124, z = −1.195, *p* = 0.232 (Table 7; Figure 1b). 

At the average mediated effects across levels (mean level) of neuroticism (Table 7), family climate partially mediated the effect of individual impact on anxiety (IE = 0.081, CI = [0.018, 0.144], beta= 0.027, z = 2.52, *p* = 0.012). Moreover, the single components of the indirect path, the direct and total mediation were significant. 

For high levels of neuroticism (mean + SD), the mediated effect of family climate to predict anxiety was significant, but without showing either a larger or smaller moderator impact compared to the mean level of neuroticism (IE = 0.127, CI = [0.028, 0.225], beta = −0.042, z = 2.53, *p* = 0.012). Instead, the direct mediation of Individual Impact to Anxiety showed a larger effect (IE = 1.052, CI = [0.686, 1.418], beta = 0.352, z = 5.63, *p* < 0.001).

For low levels of neuroticism (mean-SD), the mediated effect of family climate to predict depression did not maintain the significance (IE = 0.044, CI = [−0.006, 0.094], beta = 0.015, z = 1.74, *p* = 0.082). Instead, the direct mediation of individual impact to depression showed a smaller effect compared to the mean and high levels of neuroticism (IE = 0.307, CI = [0.019, 0.560], beta = 0.104, z = 2.09, *p* = 0.037).

*Stress*. Neuroticism did not moderate the direct and indirect paths in a substantial way, including with respect to the single components of the indirect path (Table 8; Figure 1c). 

At the average mediated effects across levels (mean level) of neuroticism, family climate did not mediate the effect of individual impact on stress, and individual impact totally predicted stress (IE = 0.710, CI = [0.485, 0.935], beta = 0.273, z = 6.185, *p* < 0.001). Moreover, the single component of the indirect path from individual impact to family climate and the total mediation were significant (Table 8). 

For high levels of neuroticism (mean + SD), the partially mediated effect of family climate to predict stress was significant (IE = 0.072, CI = [0.005, 0.139], beta = 0.027, z = 2.104, *p* = 0.035). Instead, the direct mediation of individual impact to anxiety showed a slightly larger moderation effect (IE = 0.843, CI = [0.519, 1.167], beta = 0.323, z = 5.105, *p* < 0.001). Moreover, the single components of the indirect path and total mediation were significant. 

For low levels of neuroticism (mean-SD), the mediated effect of family climate to predict depression did not maintain the significance. instead, the direct mediation of individual impact to depression showed a smaller moderation effect compared to the mean and high levels of neuroticism (IE = 0.577, CI = [0.322, 0.832], beta = 0.222, z = 6.185, *p* < 0.001).

## 4. Discussion

The overall goal of this study was to evaluate the psychosocial correlates of the COVID-19 pandemic on Italian LGBT+ young adults to identify potential individual and contextual factors as predictors of less adverse mental health conditions. Preliminarily, we assessed the psychosocial outcomes of the COVID-19 pandemic on Italian LGBT+ young adults, exploring correlations between psychological symptoms (depression, anxiety, stress), individual impact of the COVID-19 pandemic (family climate, perceived degree of social isolation), the individual personality tendency toward negative feelings (e.g., neuroticism), and enacted stigma. We found a significant association between negative attitudes towards young adults’ LGBT+ identity within the family context and internalizing symptoms of distress. These findings are in line with literature published before [96,97] and during the COVID-19 pandemic [40,41,42,43,44,45,84], further highlighting even more the fundamental role of the family environment during pandemic restrictions as a potential risk factor for young people of sexual and gender minorities [76,77,78,79]. Despite no statistically significant differences in distress having been reported between participants living with their own family and in different configurations (e.g., living alone or with roommates), the latter reported overall more feelings of freedom in expressing their LGBT+ identity. Finally, we also found an association between minority stress and adverse mental health outcomes, further corroborating the existing literature on the topic [34,35,98,99,100,101].

As our first aim, the factors that consistently and meaningfully predicted all DASS-21 scores were the individual impact of the COVID-19 pandemic and family climate. The individual impact of the COVID-19 pandemic as a predictor was expected, as the COVID-19 health emergency taxed the general population, decreasing the psychophysical well-being of people worldwide [6,7,8]. The results indicate that family climate (related to participants’ LGBT+ status during lockdown restrictions) partially mediated the relationship between the impact of the COVID-19 pandemic on an individual level and mental health conditions in the form of depression and anxiety.

Regarding the second aim, we also found that the personality trait of neuroticism significantly moderated the strength of the relationship between LGBT+ status during lockdown restrictions and internalizing symptoms. Over the years, psychological research has extensively documented that the individual disposition of traits to experience negative and adverse effects may be more associated with lower psychological well-being [49,50,102].

Our results also suggest that, in the presence of lower and medium neuroticism, the family climate mediates the relationship between individual impact and depression. In the presence of higher neuroticism, the family climate loses its mediating effect, and the role of personality traits emerges as being decisive. Regarding the relationship between individual impact and anxiety, the family climate mediating their relationship emerges as being decisive. 

Our findings contribute to furthering our understanding of the role of neuroticism in the impact on the psychological well-being of LGBT+ young people and, presumably, the general population, during a global health emergency represented by the COVID-19 pandemic. In fact, although research so far has focused on the cisgender and heterosexual population, there is no reason to conclude that the explanatory power of trait disposition to experience negative emotions in terms of mental health outcomes would be different for sexual and gender minorities, particularly those belonging to Western cultural groups, where Big Five Personality Theory has been most studied.

### Limitations and Further Studies

Our study is not exempt from limitations. First, non-probability convenience sampling limits the generalizability of our findings to the population at large. Indeed, our participants were on average highly educated, and resided predominantly in urban areas of Northern Italy. Furthermore, the length of the questionnaire (around 20 min) and its exclusively online administration may have led to self-selection bias. Additionally, although we chose a nonparametric approach, the unequal sample size for the intersex, transgender, non-binary, and pansexual and “other” sexual and gender minority groups poses concerns regarding the robustness of the statistical analyses, particularly with respect to those carried out in order to explore differences among participants based on sex assigned at birth, gender identity, and sexual orientation. Future research should include a larger number of intersex, transgender, gender variant, and alternative sexual minority people, and evaluate the psychosocial effects of the COVID-19 pandemic related to the specific needs of various marginalized groups. In this regard, as in previous studies [40,41,83,84] our research study applied an approach that did not consider internal differences in the LGBT+ group. This aspect represents a limitation that requires further investigation using a multi-method approach (e.g., qualitative investigations; observational methods) in order bring out the specific challenges and needs of specific groups. 

Nevertheless, our work also presents various strengths. First, the overall sample size was moderately large, also thanks to the numerous local and national LGBT+ associations that contributed to recruitment; as a consequence, a wide variety of sexual and gender minority identities were represented. Furthermore, to the best of our knowledge, this is the first study exploring the role of neuroticism in moderating psychological distress symptoms in sexual and gender minorities. 

## 5. Conclusions

In conclusion, the present findings support the need for large scale interventions aimed at reducing negative family attitudes towards sexual and gender minority identities and promoting understanding and acceptance, as perceived family support in relation to LGBT+ status may play a role in putting young adults at increased risk for worse mental health conditions following the impact of the COVID-19 pandemic at the individual level. The implications in clinical terms have been discussed; a brief mention of some social considerations is equally important. The context of aggregation and exchange with other LGBT+ people is a crucial protective factor [103], even more so in specific moments of individual development. The pandemic, and the social restrictions associated with it, restricted exploration of identity in confrontation with others, limiting this aspect to a solitary context within stigmatizing contexts as well. The social–political context must recognize this important role for LGBT+ associations. Conversely, associations must recognize their importance by promoting projects aimed at the most fragile segment of LGBT+ population by using online devices not only as dissemination tools but also by finding new spaces of aggregation.

## Figures and Tables

**Table 1:** Sociodemographic characteristics of participants.

Variable	*n*	%	Variable	*n*	%
***Sex assigned at birth***			***Area of residence***		
Female	228	56.02	Rural	110	27.03
Male	171	42.01	Urban	297	72.97
Intersex	3	0.74	***Relationship status***		
Prefer not to say	5	1.23	Not in a relationship	190	46.68
***Gender identity***			In a relationship	217	53.32
Cisgender women	170	41.77	***Living with family***		
Cisgender men	150	36.86	Yes	277	68.06
Transgender persons	16	3.93	No	130	31.94
Non-binary persons	51	12.53	***Educational level***		
Other	21	5.16	Lower secondary	25	6.14
***Sexual orientation***			Upper secondary	211	51.84
Gay/lesbian	230	56.51	Bachelor’s	89	21.87
Bisexual	104	25.55	Postgraduate	82	20.15
Pansexual	40	9.83	***Work status***		
Asexual	9	2.21	Student	172	42.26
Heterosexual	5	1.23	Student-worker	47	11.55
Other	19	4.67	Full-time worker	92	22.60
***Nationality***			Part-time worker	32	7.86
Italian	397	97.54	Unemployed	49	12.04
Other	10	2.46	Payroll subsidies	1	0.25
***Region of residence***			Other	14	3.44
North	261	64.13	***Work changes due to COVID-19***		
Centre	69	16.95	Yes	161	39.56
South	52	12.78	No	246	60.44
Islands	25	6.14			

**Table 2:** Results of the Second EFA for Psychosocial Effects of the COVID-19 Pandemic items.

	Factor Loading				
	1	2	3	Communality	Uniqueness
Item 1	**0.71**	0.20		0.55	0.45
Item 2	**0.73**	0.17		0.58	0.42
Item 3	**0.76**	0.16	0.18	0.64	0.36
Item 6	0.35	0.20	**0.53**	0.44	0.56
Item 9	0.31	**0.68**	0.11	0.57	0.43
Item 10	0.18	**0.97**	0.12	0.99	0.01
Item 7	0.17	0.11	**0.79**	0.67	0.33
Item 8			**0.72**	0.51	0.49
% Explained variance	0.24	0.20	0.19		
Eigenvalue	1.90	1.56	1.49		
Coefficient alpha	0.81	0.73	0.84		

*Note*. n = 407. ‘Maximum likelihood’ extraction method was used in combination with a ‘varimax’ rotation. Item 8 was reverse scored before the analysis.

**Table 3:** Descriptive Statistics for the Psychosocial Effects of the COVID-19 scale, DASS-21, NEO-FFI-3 Neuroticism and MSS Enacted stigma for the entire group of participants.

Variable	Mean	SD	Median	MAD	Min	Max	Skew	Kurtosis	SE
Individual Impact	7.24	2.06	7.67	1.98	0	10	−0.88	0.32	0.10
Social Isolation	5.93	3.07	6.50	3.71	0	10	−0.54	−0.74	0.15
Family Climate	6.55	2.67	7.00	2.97	0	10	−0.57	−0.61	0.13
DASS_Depression	10.81	6.39	11	8.90	0	21	0.06	−1.21	0.32
DASS_Anxiety	7.51	6.11	6	5.93	0	21	0.60	−0.85	0.30
DASS_Stress	12.91	5.35	13	5.93	0	21	−0.23	−0.94	0.27
NEO-FFI3_Neuroticism	72.88	14.89	74.32	15.44	25.34	102.59	−0.26	−0.52	0.74
MSS_Enacted stigma	2.11	0.76	2	0.74	1	5	0.60	0.05	0.04

**Table 4:** Multiple Regressions for Depression, Anxiety and Stress as dependent variables.

	M1 Depression							M2 Anxiety							M3 Stress						
						**95% C.I.**							**95% C.I.**							**95% C.I.**	
**Predictor (model 1)**	**Estimate**	**SE**	**t**	***p***	**Stand. Estimate**	**Lower**	**Upper**	**Estimate**	**SE**	**t**	***p***	**Stand. Estimate**	**Lower**	**Upper**	**Estimate**	**SE**	**t**	***p***	**Stand. Estimate**	**Lower**	**Upper**
Intercept ^a^	6.534	2.183	2.993	0.003				5.064	2.093	2.420	0.016				9.746	1.921	5.073	<0 .001			
Age	−0.131	0.078	−1.670	0.096	−0.096	−0.209	0.017	−0.235	0.075	−3.126	0.002	−0.180	−0.293	−0.067	−0.154	0.069	−2.228	0.026	−0.134	−0.253	−0.016
Gender Identity																					
other–cis	0.509	1.306	0.390	0.697	0.080	−0.322	0.481	−0.937	1.252	−0.749	0.455	−0.153	−0.556	0.249	1.680	1.149	1.462	0.145	0.314	−0.108	0.736
nb–cis	0.496	0.908	0.546	0.585	0.078	−0.202	0.357	−0.524	0.870	−0.603	0.547	−0.086	−0.366	0.194	0.416	0.799	0.521	0.602	0.078	−0.216	0.371
tg–cis	−1.346	1.543	−0.872	0.384	−0.211	−0.685	0.264	−1.249	1.479	−0.844	0.399	−0.204	−0.680	0.272	−1.325	1.358	−0.976	0.330	−0.248	−0.747	0.251
Sexual Orientation:																					
other–homo	0.374	1.132	0.330	0.742	0.058	−0.290	0.407	0.202	1.085	0.186	0.852	0.033	−0.316	0.382	−0.106	0.996	−0.106	0.915	−0.020	−0.386	0.346
bisex–homo	1.677	0.710	2.364	0.019	0.263	0.044	0.481	0.487	0.680	0.716	0.474	0.080	−0.139	0.299	1.172	0.625	1.876	0.061	0.219	−0.011	0.448
pan–homo	1.893	1.038	1.823	0.069	0.296	−0.023	0.616	1.685	0.995	1.692	0.091	0.276	−0.045	0.596	1.407	0.914	1.540	0.124	0.263	−0.073	0.599
Living in Family	0.753	0.661	1.140	0.255	0.055	−0.040	0.150	0.315	0.634	0.497	0.619	0.024	−0.071	0.119	−0.153	0.582	−0.264	0.792	−0.013	−0.113	0.086
Education	0.035	0.637	0.055	0.957	0.003	−0.094	0.100	−0.065	0.611	−0.106	0.915	−0.005	−0.102	0.092	0.335	0.561	0.598	0.551	0.031	−0.071	0.133
Employment Situation	−1.810	0.655	−2.763	0.006	−0.141	−0.242	−0.041	0.147	0.628	0.234	0.815	0.012	−0.089	0.113	−0.018	0.577	−0.031	0.976	−0.002	−0.107	0.104
In a Romantic Relationship	−0.348	0.595	−0.584	0.559	−0.027	−0.119	0.064	−0.119	0.571	−0.208	0.835	−0.010	−0.101	0.082	0.544	0.524	1.039	0.299	0.051	−0.045	0.147
other problems:																					
pnr–no	3.281	0.930	3.528	<0 .001	0.513	0.227	0.800	3.153	0.891	3.537	<0 .001	0.516	0.229	0.803	2.677	0.818	3.271	0.001	0.500	0.200	0.801
yes–no	3.901	0.656	5.943	<0 .001	0.610	0.409	0.813	4.079	0.629	6.482	<0 .001	0.668	0.465	0.870	2.564	0.578	4.438	<0 .001	0.479	0.267	0.691
Enacted Stigma	1.528	0.389	3.924	<0 .001	0.183	0.091	0.274	1.693	0.373	4.536	<0 .001	0.212	0.120	0.304	1.108	0.343	3.233	0.001	0.158	0.062	0.255
Time	1.399	0.631	2.216	0.027	0.102	0.012	0.192	1.574	0.605	2.601	0.010	0.120	0.029	0.210	1.341	0.556	2.414	0.016	0.116	0.022	0.211
						**95% C.I.**							**95% C.I.**							**95% C.I.**	
**Predictor (model 2)**	**Estimate**	**SE**	**t**	***p***	**Stand. Estimate**	**Lower**	**Upper**	**Estimate**	**SE**	**t**	***p***	**Stand. Estimate**	**Lower**	**Upper**	**Estimate**	**SE**	**t**	***p***	**Stand. Estimate**	**Lower**	**Upper**
Intercept ^a^	−7.898	2.324	−3.399	<0 .001				−3.255	2.442	−1.333	0.183				−0.444	2.192	−0.202	0.840			
Age	−0.020	0.063	−0.315	0.753	−0.014	−0.105	0.076	−0.183	0.066	−2.765	0.006	−0.140	−0.239	−0.040	−0.099	0.059	−1.663	0.097	−0.086	−0.188	0.016
Gender Identity																					
other–cis	0.123	1.033	0.119	0.905	0.019	−0.299	0.337	−1.507	1.085	−1.389	0.166	−0.247	−0.596	0.103	1.208	0.974	1.240	0.216	0.226	−0.132	0.584
nb–cis	−0.369	0.719	−0.513	0.608	−0.058	−0.279	0.164	−1.263	0.755	−1.672	0.095	−0.207	−0.450	0.036	−0.201	0.678	−0.297	0.767	−0.038	−0.287	0.212
tg–cis	−1.740	1.219	−1.427	0.154	−0.272	−0.647	0.103	−1.446	1.281	−1.129	0.260	−0.237	−0.649	0.176	−1.406	1.150	−1.222	0.222	−0.263	−0.685	0.160
Sexual Orientation:																					
other–homo	0.715	0.899	0.795	0.427	0.112	−0.165	0.389	0.681	0.945	0.720	0.472	0.111	−0.193	0.415	0.473	0.848	0.558	0.578	0.088	−0.223	0.400
bisex–homo	0.541	0.564	0.959	0.338	0.085	−0.089	0.258	−0.329	0.593	−0.556	0.579	−0.054	−0.245	0.137	0.355	0.532	0.667	0.505	0.066	−0.129	0.262
pan–homo	0.786	0.822	0.956	0.340	0.123	−0.130	0.376	0.870	0.864	1.007	0.315	0.142	−0.136	0.420	0.682	0.776	0.879	0.380	0.127	−0.158	0.412
Living in Family	0.977	0.537	1.820	0.070	0.071	−0.006	0.149	0.563	0.564	0.997	0.319	0.043	−0.042	0.128	−0.162	0.507	−0.320	0.749	−0.014	−0.101	0.073
Education	−0.170	0.502	−0.338	0.736	−0.013	−0.090	0.063	−0.245	0.528	−0.463	0.643	−0.020	−0.104	0.064	0.166	0.474	0.349	0.727	0.015	−0.071	0.101
Employment Situation	−0.886	0.522	−1.695	0.091	−0.069	−0.149	0.011	0.895	0.549	1.630	0.104	0.073	−0.015	0.161	0.717	0.493	1.455	0.146	0.067	−0.024	0.157
In a Romantic Relationship	−0.144	0.479	−0.301	0.763	−0.011	−0.085	0.062	0.265	0.503	0.526	0.599	0.022	−0.059	0.102	0.719	0.451	1.593	0.112	0.067	−0.016	0.150
other problems:																					
pnr–no	1.008	0.748	1.346	0.179	0.158	−0.073	0.388	1.504	0.786	1.912	0.057	0.246	−0.007	0.499	1.092	0.706	1.547	0.123	0.204	−0.055	0.463
yes–no	1.562	0.545	2.868	0.004	0.244	0.077	0.412	2.389	0.572	4.175	<0 .001	0.391	0.207	0.575	0.834	0.514	1.624	0.105	0.156	−0.033	0.345
Enacted Stigma	0.013	0.329	0.038	0.969	0.002	−0.076	0.079	0.302	0.346	0.873	0.383	0.038	−0.047	0.123	−0.154	0.311	−0.497	0.620	−0.022	−0.109	0.065
Time	0.186	0.532	0.350	0.727	0.014	−0.063	0.090	0.340	0.559	0.607	0.544	0.026	−0.058	0.110	−0.012	0.502	−0.023	0.982	−9.95 × 10^−4^	−0.087	0.085
Individual Impact	0.460	0.140	3.288	0.001	0.148	0.060	0.237	0.451	0.147	3.065	0.002	0.152	0.054	0.249	0.619	0.132	4.691	<0 .001	0.238	0.138	0.338
Family Climate	−0.286	0.102	−2.815	0.005	−0.120	−0.203	−0.036	−0.360	0.107	−3.365	<0 .001	−0.157	−0.249	−0.065	−0.167	0.096	−1.742	0.082	−0.083	−0.178	0.011
Social Isolation	−0.094	0.085	−1.101	0.272	−0.045	−0.126	0.035	0.133	0.090	1.486	0.138	0.067	−0.022	0.155	0.066	0.080	0.818	0.414	0.038	−0.053	0.128
Neuroticism	0.228	0.019	11.993	<0 .001	0.530	0.443	0.617	0.141	0.020	7.069	<0 .001	0.344	0.248	0.439	0.145	0.018	8.077	<0 .001	0.402	0.304	0.500

^a^ Represents reference level.

**Table 5:** Multiple Linear Regression Models’ Goodness-of-fit Measures and Comparison in Predicting DASS-21 Depression, Anxiety, Stress, and General Distress Scores.

Overall Model Test								
	Model	R	R²	adj. R²	F	df1	df2	*p*
** Depression **								
	Model fit measures							
	1	0.495	0.245	0.216	8.45	15	391	< 0.001
	2	0.732	0.537	0.514	23.58	19	387	< 0.001
		Comparison						
		*Model*		Δ*R^2^*	*F*	*df1*	*df2*	*p*
		1	2	0.292	60.9	4	387	< 0.001
** Anxiety **								
	Model fit measures							
	1	0.491	0.241	0.212	8.29	15	391	< 0.001
	2	0.664	0.441	0.413	16.04	19	387	< 0.001
		Comparison						
		1	2	0.199	34.5	4	387	< 0.001
** Stress **								
	Model fit measures							
	1	0.408	0.166	0.134	5.2	15	391	< 0.001
	2	0.642	0.412	0.383	14.27	19	387	< 0.001
		Comparison						
		1	2	0.246	40.5	4	387	< 0.001

**Table 6:** Moderation Estimates with Neuroticism as a moderator (Depression = dependent variable). The tables show the moderation effects (interactions) and the conditional mediation. Confidence intervals were computed with method standard (Delta method).

**Moderator**	**Interaction**		**Estimate**	**SE**	**Lower**	**Upper**	**β**	**z**	***p***
neuroticism	neuroticism:individual_impact ⇒ family_climate		−0.003	0.004	−0.010	0.004	−0.036	−0.781	0.435
	neuroticism:individual_impact ⇒ depression		0.018	0.007	0.004	0.031	0.093	2.538	0.011
	neuroticism:family_climate ⇒ depression		0.007	0.006	−0.005	0.018	0.110	1.174	0.240
**Moderator levels**					**95% C.I. (a)**				
**neuroticism**	**Type**	**Effect**	**Estimate**	**SE**	**Lower**	**Upper**	**β**	**z**	***p***
Mean-1·SD	Indirect	individual_impact ⇒ family_climate ⇒ depression	0.075	0.033	0.010	0.140	0.024	2.250	0.025
Mean-1·SD	Component	individual_impact ⇒ family_climate	−0.201	0.075	−0.347	−0.055	−0.155	−2.700	0.007
Mean-1·SD		family_climate ⇒ depression	−0.372	0.092	−0.552	−0.193	−0.155	−4.070	<0.001
Mean-1·SD	Direct	individual_impact ⇒ depression	0.253	0.139	−0.020	0.525	0.081	1.820	0.069
Mean-1·SD	Total	individual_impact ⇒ depression	0.341	0.139	0.068	0.614	0.110	2.450	0.014
Mean	Indirect	individual_impact ⇒ family_climate ⇒ depression	0.066	0.028	0.010	0.121	0.021	2.310	0.021
Mean	Component	individual_impact ⇒ family_climate	−0.244	0.065	−0.371	−0.116	−0.188	−3.740	<0.001
Mean		family_climate ⇒ depression	−0.270	0.092	−0.449	−0.090	−0.113	−2.950	0.003
Mean	Direct	individual_impact ⇒ depression	0.516	0.123	0.276	0.756	0.166	4.210	<0.001
Mean	Total	individual_impact ⇒ depression	0.587	0.122	0.348	0.826	0.189	4.820	<0.001
Mean+1·SD	Indirect	individual_impact ⇒ family_climate ⇒ depression	0.048	0.031	−0.012	0.108	0.015	1.560	0.118
Mean+1·SD	Component	individual_impact ⇒ family_climate	−0.286	0.094	−0.471	−0.101	−0.220	−3.030	0.002
Mean+1·SD		family_climate ⇒ depression	−0.167	0.092	−0.347	0.012	−0.070	−1.830	0.068
Mean+1·SD	Direct	individual_impact ⇒ depression	0.780	0.176	0.434	1.125	0.251	4.420	<0.001
Mean+1·SD	Total	individual_impact ⇒ depression	0.833	0.176	0.488	1.179	0.268	4.720	<0.001

**Table 7:** Moderation Estimates with Neuroticism as a moderator (Anxiety = dependent variable). The tables show the moderation effects (interactions) and the conditional mediation. Confidence intervals were computed with method standard (Delta method).

**Moderator**	**Interaction**		**Estimate**	**SE**	**Lower**	**Upper**	**β**	**z**	***p***
neuroticism	neuroticism:individual_impact ⇒ family_climate		−0.003	0.004	−0.010	0.004	−0.036	−0.781	0.435
	neuroticism:individual_impact ⇒ anxiety		0.025	0.007	0.011	0.039	0.138	3.389	<0.001
	neuroticism:family_climate ⇒ anxiety		−0.007	0.006	−0.020	0.005	−0.124	−1.195	0.232
**Moderator levels**					**95% C.I. (a)**				
**neuroticism**	**Type**	**Effect**	**Estimate**	**SE**	**Lower**	**Upper**	**β**	**z**	***p***
Mean-1·SD	Indirect	individual_impact ⇒ family_climate ⇒ anxiety	0.044	0.026	−0.006	0.094	0.015	1.740	0.082
Mean-1·SD	Component	individual_impact ⇒ family_climate	−0.201	0.075	−0.347	−0.055	−0.155	−2.700	0.007
Mean-1·SD		family_climate ⇒ anxiety	−0.221	0.097	−0.411	−0.031	−0.097	−2.280	0.023
Mean-1·SD	Direct	individual_impact ⇒ anxiety	0.307	0.147	0.019	0.596	0.104	2.090	0.037
Mean-1·SD	Total	individual_impact ⇒ anxiety	0.338	0.149	0.046	0.629	0.114	2.270	0.023
Mean	Indirect	individual_impact ⇒ family_climate ⇒ anxiety	0.081	0.032	0.018	0.144	0.027	2.520	0.012
Mean	Component	individual_impact ⇒ family_climate	−0.244	0.065	−0.371	−0.116	−0.188	−3.740	<0.001
Mean		family_climate ⇒ anxiety	−0.332	0.097	−0.522	−0.142	−0.145	−3.420	<0.001
Mean	Direct	individual_impact ⇒ anxiety	0.680	0.130	0.425	0.934	0.229	5.240	<0.001
Mean	Total	individual_impact ⇒ anxiety	0.755	0.130	0.500	1.010	0.254	5.810	<0.001
Mean+1·SD	Indirect	individual_impact ⇒ family_climate ⇒ anxiety	0.127	0.050	0.028	0.225	0.042	2.530	0.012
Mean+1·SD	Component	individual_impact ⇒ family_climate	−0.286	0.094	−0.471	−0.101	−0.220	−3.030	0.002
Mean+1·SD		family_climate ⇒ anxiety	−0.442	0.097	−0.632	−0.252	−0.192	−4.560	<0001
Mean+1·SD	Direct	individual_impact ⇒ anxiety	1.052	0.187	0.686	1.418	0.352	5.630	<0.001
Mean+1·SD	Total	individual_impact ⇒ anxiety	1.172	0.188	0.803	1.541	0.394	6.230	<0.001

**Table 8:** Moderation Estimates with Neuroticism as a moderator (Stress = dependent variable). The tables show the moderation effects (interactions) and the conditional mediation. Confidence intervals were computed with method standard (Delta method).

**Moderator**	**Interaction**		**Estimate**	**SE**	**Lower**	**Upper**	**β**	**z**	***p***
neuroticism	neuroticism:individual_impact ⇒ family_climate		−0.003	0.004	−0.010	0.004	−0.036	−0.781	0.435
	neuroticism:individual_impact ⇒ stress		0.009	0.007	−0.004	0.022	0.056	1.369	0.171
	neuroticism:family_climate ⇒ stress		−0.008	0.006	−0.019	0.003	−0.149	−1.422	0.155
**Moderator levels**					**95% C.I. (a)**				
**neuroticism**	**Type**	**Effect**	**Estimate**	**SE**	**Lower**	**Upper**	**Β**	**z**	***p***
Mean-1·SD	Indirect	individual_impact ⇒ family_climate ⇒ stress	0.004	0.017	−0.030	0.038	0.001	0.207	0.836
Mean-1·SD	Component	individual_impact ⇒ family_climate	−0.201	0.075	−0.347	−0.055	−0.155	−2.698	0.007
Mean-1·SD		family_climate ⇒ stress	−0.018	0.086	−0.186	0.150	−0.009	−0.208	0.835
Mean-1·SD	Direct	individual_impact ⇒ stress	0.577	0.130	0.322	0.832	0.222	4.430	<0.001
Mean-1·SD	Total	individual_impact ⇒ stress	0.566	0.130	0.311	0.820	0.217	4.354	<0.001
Mean	Indirect	individual_impact ⇒ family_climate ⇒ stress	0.033	0.023	−0.012	0.077	0.013	1.443	0.149
Mean	Component	individual_impact ⇒ family_climate	−0.244	0.065	−0.371	−0.116	−0.188	−3.736	<0.001
Mean		family_climate ⇒ stress	−0.134	0.086	−0.302	0.034	−0.067	−1.565	0.118
Mean	Direct	individual_impact ⇒ stress	0.710	0.115	0.485	0.935	0.273	6.185	<0.001
Mean	Total	individual_impact ⇒ stress	0.737	0.114	0.514	0.960	0.283	6.481	<0.001
Mean+1·SD	Indirect	individual_impact ⇒ family_climate ⇒ stress	0.072	0.034	0.005	0.139	0.027	2.104	0.035
Mean+1·SD	Component	individual_impact ⇒ family_climate	−0.286	0.094	−0.471	−0.101	−0.220	−3.032	0.002
Mean+1·SD		family_climate ⇒ stress	−0.251	0.086	−0.419	−0.083	−0.124	−2.922	0.003
Mean+1·SD	Direct	individual_impact ⇒ stress	0.843	0.165	0.520	1.167	0.323	5.105	<0.001
Mean+1·SD	Total	individual_impact ⇒ stress	0.908	0.165	0.586	1.231	0.349	5.518	<0.001

## Data Availability

Restrictions apply to the availability of these data. Data are available from the Principal Investigators (MM and PR) upon reasonable request.
